# The role of CA-125, GLS and FASN in predicting cytoreduction for epithelial ovarian cancers

**DOI:** 10.1186/s13104-020-05188-x

**Published:** 2020-07-22

**Authors:** G. N. A. Winarno, Y. M. Hidayat, S. Soetopo, S. R. Krisnadi, M. D. L. Tobing, S. Rauf

**Affiliations:** 1 Department of Obstetrics and Gynecology, Faculty of Medicine, Padjadjaran University, Jl Pasteur No.38, Bandung, Indonesia; 2 Department of Radiology, Faculty of Medicine, Padjadjaran University, Jl Pasteur No.38, Bandung, Indonesia; 3 Department of Obstetrics and Gynecology, Faculty of Medicine, Hasanuddin University, Jl. Perintis Kemerdekaan KM. 10, Makasar, Indonesia

**Keywords:** Epithelial ovarian cancer, CA-125, FASN, GLS, Cytoreductive surgery

## Abstract

**Objective:**

Cytoreduction has an important role in improving the survival rate of epithelial ovarian cancer (EOC) patients. This study aimed to assess the ability of preoperative serum CA125, FASN and GLS as predictors of cytoreductive surgery for epithelial ovarian cancer (EOC).

**Results:**

The average values of serum CA-125, FASN, and GLS in the suboptimal cytoreduction group were higher than those in optimal cytoreduction group. The cut off point (COP) was 248.55 (*p* = 0.0001) with 73.2% sensitivity and 73.6% specificity for CA-125, 0.445 (*p* = 0.017) with 62.5% sensitivity and 60.4% specificity for FASN, and 22.895 (*p* = 0.0001) with 73.2% sensitivity and 75.5% specificity for GLS. The COP of CA-125 and GLS combined was 29.16 (*p* = 0.0001) with sensitivity 82.1% and specificity 73.6%, while the COP of CA-125, GLS, and FASN combined was 0.83 (*p* = 0.0001) with 87.5% sensitivity and 73.6% specificity.

## Introduction

With the recent advancements in science and technology, therapies for ovarian cancer is also continue to be developed. The management of ovarian cancer cases has developed in terms of surgical technology and chemotherapeutic regimens [1]. In evaluating the therapeutic response to treatment in ovarian cancer, clinicians continue to develop new drugs with targets related to cancer cell proliferation [1].

Despite many improvements in therapy for ovarian cancer patients, a study by Zhang Xi et al. study in China that compared the survival rate of EOC (epithelial ovarian cancer) between the September 1998-August 2007 and September 2007-August 2012 periods found only a meagre increase from 59.2 to 63% [2]. The latest report from the American Cancer Society states that the survival rate of EOC for the period 2009–2015 was 47% based on data on the incidence and trends of cancer in the United States [3].

Ovarian cancer is often referred to as the “Silent Killer” because most patients are already in an advanced stage upon diagnosis due to the lack of effective screening measures. The standard treatment for advanced stage EOC is cytoreductive surgery followed by adjuvant first-line platinum-taxane chemotherapy. Optimal cytoreduction is achieved with residual tumour of less than 1 cm, while suboptimal cytoreduction is achieved with the residual tumour of more than 1 cm [4].

Several studies have shown a significant increase in survival rates in patients who have undergone optimal cytoreduction [5–7]. In a study conducted by Bacalbasa in 2015 with 99 patients with stage IIIC-IV ovarian cancer, the average survival duration of those who underwent optimal cytoreduction surgery was 72 weeks, while among patients who underwent suboptimal cytoreduction surgery, the duration was 51 weeks [8].

Optimal cytoreduction is the most convincing method of increasing survival time in patients with ovarian cancer [9]. However, additional exams such as computed tomography (CT) and magnetic resonance imaging (MRI) are required to determine whether a patient is suitable for optimal cytoreduction. This has been the chief obstacle in providing optimal cytoreduction to ovarian cancer patients in Indonesia, where neither diagnostic modality is always available or affordable. Therefore, a serum biomarker that could predict the outcome of cytoreduction surgery in ovarian cancer could make the procedure more accessible in the absence of CT and MRI.

Fatty acid synthase (FASN) supports cancer cells to resist oxidative stress and limits the absorption of chemotherapy drugs [10, 11]. There is a relationship between the stage of ovarian cancer and FASN levels; in one study, the level of FASN in end-stage cancer patients was 94.1%, while in patients with stage I ovarian cancer, it was 12.5% [12].

Glutaminase (GLS) is an enzyme needed for the process of glutaminolysis which converts glutamine to glutamate.[13] Glutamine is an amino acid needed to support the growth of cancer cells and the formation of ATP.[14] In ovarian cancer with a high invasion rate, the rate of glutaminolysis will be higher than in ovarian cancer with a lower invasiveness.[15] This study assessed the use of preoperative serum CA-125, FASN, and GLS levels as predictors of cytoreduction in EOC, both individually and in combination.

## Material and methods

The records of RSUP Dr. Hasan Sadikin Bandung were utilized to identify all patients with suspected EOC who underwent primary cytoreductive surgery from 2017 until 2019. This study was an observational analytic study with a cross-sectional design. The inclusion criteria in this study were ovarian cancer patients who underwent primary cytoreductive surgery, did not suffer from chronic diseases or other tumours, and were willing to participate in the study after completing informed consent forms. Patients who did not have epithelial-type ovarian cancer stage III-IV based on histopathology examination or with suboptimal histopathological preparations and blood serum were excluded in this study.

Preoperative serum FASN, GLS, and CA-125 levels, were independent variables while cytoreduction surgery, both optimal and suboptimal, was the dependent variable in this study. The cut-off point (COP) was determined using receiver operating characteristic (ROC) curve 23 analysis. The statistical analyses were performed with SPSS™ (24.0.0).

The distributions of the variables were assed for normality with the Kolmogorov–Smirnov test. Characteristics between the 2 groups were compared with the unpaired t-test (normally distributed) and the Mann–Whitney U test (non-normally distributed). Categorical data were compared with the Chi-squared test or, alternatively, Fisher's Exact test or and Kolmogorov–Smirnov test.

Blood samples were taken from the medial cubital vein (volume 1 mL) before cytoreductive surgery. The blood samples of patients who had been confirmed by histopathological examination as EOC patients were examined using the enzyme-linked immunosorbent assay (ELISA) method at the Laboratory of Molecular Genetics of Padjajaran University.

Samples collected were left for 2 h at room temperature or overnight at 4 °C prior to centrifugation the next day, after which they were stored at − 20 °C or − 80 °C for later use. CA-125, FASN and GLS were assessed by ELISA kits (ADVIA Centaur CA125II, Siemens, United States; SEC470Hu, Cloud-Clone, United States, and SEJ026Hu, Cloud-Clone, United States, respectively) and prepared according to the manufacturer’s instructions.

## Results

### Characteristics of the sample

One hundred nine patients were diagnosed with EOC during the study period. Most types of EOC were mucinous (34.9%). 53 patients underwent suboptimal cytoreductive surgery, while 56 patients underwent optimal cytoreduction (Table 1).

**Table 1:** Background characteristics of the study population

Variable	N = 109	Group		*P* value
		Suboptimal cytoreduction	Optimal cytoreduction	
		N = 56	N = 53	
Age (years)				*0.269*
Mean ± Std	47.48 ± 11.347	48.66 ± 9.648	46.24 ± 12.880	
Median	47.00	47.00	47.00	
Range (min–max)	17/00–75.00	26.00–64.00	17.00–75.00	
Parity				*0.261*
0	28 (25.7%)	16 (28.6%)	12 (22.6%)	
1	16 (14.7%)	6 (10.7%)	10 (18.9%)	
2	23 (21.1%)	15 (26.8%)	8 (15.1%)	
> 3	42 (38.5%)	19 (33.9%)	23 (43.4%)	
IMT				*0.353*
Mean ± Std	21.30 ± 3.907	20.96 ± 3.675	21.66 ± 4.143	
Median	21.30	21.25	21.40	
Ascites				*0.089*
Mean ± Std	1652.56 ± 3664.119	1703.75 ± 3393.296	1598.49 ± 3962.289	
Median	300.00	400.00	300.00	
Stage				*0.0001*
II	42 (38.5%)	8 (14.3%)	34 (64.2%)	
III	57 (52.3%)	38 (67.9%)	19 (35.8%)	
IV	10 (9.2%)	10 (17.9%)	0 (0.0%)	
Histopathology				*0.559*
Serous	27 (24.8%)	18 (32.1%)	9 (17.0%)	
Mucinous	38 (34.9%)	12 (21.4%)	26 (49.1%)	
Endometrioid	21 (19.3%)	14 (25.0%)	7 (13.2%)	
Clear cell	16 (14.7%)	8 (14.3%)	8 (15.1%)	
Others	7 (6.4%)	4 (7.1%)	3 (5.7%)	

### CA-125, FASN, and GLS serum levels

The median values of serum of CA-125, FASN, and GLS in the suboptimal cytoreduction group were higher than those in the optimal cytoreduction group. For CA-125, the median value in the suboptimal cytoreduction group was 600 U/mL, while in the optimal cytoreduction group the value was 120.30 U/mL (*p* = 0.0001), FASN had a median value of 0.50 ng/mL in the suboptimal cytoreduction group and 0.37 ng/mL in the optimal cytoreductive surgery group (*p* = 0.006), and GLS had a median value of 25.25 ng/mL in the suboptimal cytoreduction group and 20.08 ng/mL in the optimal cytoreduction group (*p* = 0.0001).

### Biomarker serum as a predictor of cytoreductive surgery

The COP values for the different substances are shown in Table 2. The COP was 248.55 for CA-125, 0.445 for FASN, 22.895 for GLS, 0.69 for CA-125 + FASN, 29.16 for CA-125 + GLS, and 0.83 for CA-125 + FASN + GLS. The formula for calculating the combined values involved multiplying the numerical value of the serum biomarker levels with the category values of the other serum biomarker levels, as described below.

**Table 2:** Comparison of means or medians between CA-125, FASN, GLS, CA-125 + FASN, CA-125 + GLS, and CA-125 + FASN + GLS serums levels for the suboptimal and optimal cytoreduction groups and cut off point, sensitivity, specificity, accuracy, positive predictive value, and negative predictive value for each variable

Variable	Group		*P* value	Cut off point	Sensitivity	Spesificity	Accuracy rate	Positive predictive value	Negative predictive value	*P* value
	Suboptimal cytoreduction	Optimal cytoreduction								
	N = 56	N = 53								
CA-125			*0.0001*	248.55	73.2%	73.6%	73.3%	74.5%	72.2%	*0.0001*
Mean ± Std	1157.62 ± 2105.195	237.52 ± 319.431								
Median	600.00	120.30								
Range (min–max)	4.29–9934.00	5.10–1941.90								
FASN			*0.006*	0.445	62.5%	60.4%	61.4%	62.5%	60.4%	*0.017*
Mean ± Std	0.58 ± 0.271	0.46 ± 0.288								
Median	0.50	0.37								
Range (min–max)	0.11–1.59	0.03–1.19								
GLS			*0.0001*	22.895	73.2%	75.5%	74.3%	75.9%	72.7%	*0.0001*
Mean ± Std	25.19 ± 5.415	20.83 ± 5.562								
Median	25.25	20.08								
Range (min–max)	14.27–37.50	10.81–38.01								
Combination CA-125 + FASN			*0.0001*	0.69	71.4%	71.7%	71.6%	72.7%	70.4%	*0.0001*
Mean ± Std	1.02 ± 0.581	0.55 ± 0.314								
Median	0.93	0.49								
Range (min–max)	0.21–3.18	0.03–1.58								
Combinantion CA-125 + GLS			*0.0001*	29.16	82.1%	73.6%	77.9%	76.6%	79.6%	*0.0001*
Mean ± Std	42.93 ± 12.913	25.83 ± 9.807								
Median	44.99	22.47								
Range (min–max)	17.15–75.00	10.81–53.52								
Combination CA-125 + FASN + GLS			*0.0001*	0.83	87.5%	73.6%	80.7%	77.8%	84.8%	*0.0001*
Mean ± Std	1.70 ± 0.967	0.66 ± 0.388								
Median	1.48	0.66								
Range (min–max)	0.22–4.68	0.03–2.16								

For the CA-125 + FASN and CA-125 + GLS combinations, the COP value was calculated using CA-125 as the category variable (1;≤248.55, 2 > 248.55) (*p* = 0.0001). The formula for the combined value of CA-125 + FASN + GLS used CA-125 (1;≤248.55, 2 > 248.55) and GLS (1;≤22.895, 2 > 22.895) as the category variables (*p* = 0.0001).

**Fig. 1:**
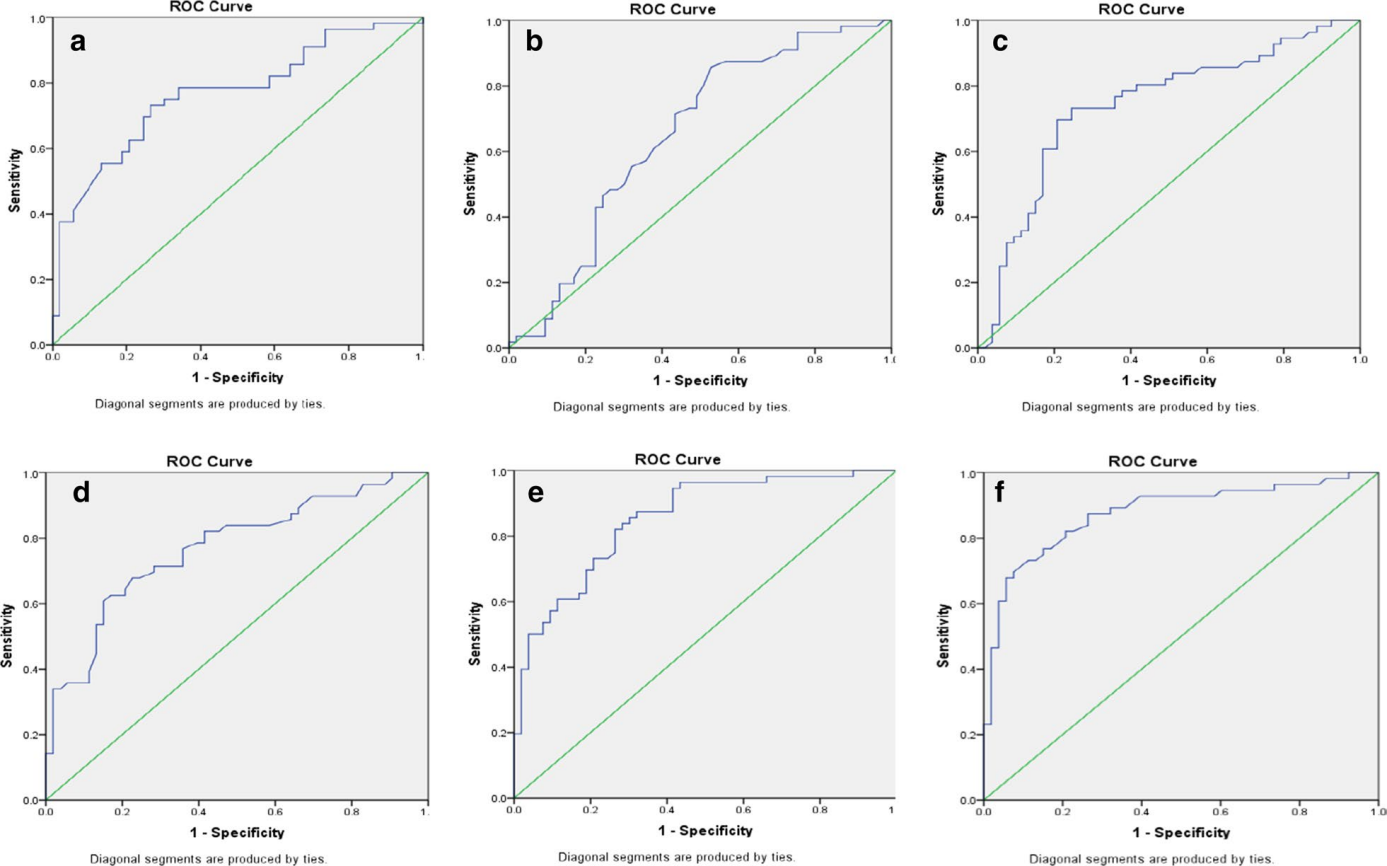
ROC curves. **a** ROC curve for the value of CA-125 in predicting cytoreduction. The AUC was 76.7% (CI 67.8–85.6%, *p* = 0.000), implying that CA-125 can predict cytoreduction correctly in 84 patients out of a total of 109 patients. **b** ROC curve for the value of FASN in predicting cytoreduction. The AUC was 65.3% (CI 54.8–75.8%, *p* = 0.006), implying that FASN can predict cytoreduction correctly in 71 patients out of a total of 109 patients. **c** ROC curve for the value of GLS in predicting cytoreduction. The AUC was 74.1% (CI 64.6–83.7%, *p* = 0.0000), implying that GLS can predict cytoreduction correctly in 81 patients out of a total of 109 patients. **d** ROC curve for the value of FASN and CA-125 in predicting cytoreduction. The AUC was 76.9% (CI 68.1–85.8%, *p* = 0.0000), implying that FASN and CA-125 can predict cytoreduction correctly in 84 patients out of a total of 109 patients. **e** ROC curve for the value of GLS and CA-125 in predicting cytoreduction. The AUC was 85.4% (CI 78.4–92.3%, *p* = 0.0000), implying that GLS and CA-125 can predict cytoreduction correctly in 91 patients out of a total of 109 patients. **f** ROC curve for the value CA-125, FASN and GLS in predicting ytoreduction. The AUC was 87.7% (CI 81–94.4%, *p* = 0.0000), implying that CA-125, FASN and GLS can predict cytoreduction correctly in 96 patients out of a total of 109 patients

The ROC curves for each of the biomarkers are presented in Fig. 1. The AUC of the ROC curve (Fig. 1) for CA-125 was 76,7% (*p* = 0.0001), for FASN was 65.3% (*p* = 0.006), for GLS was 74.1% (*p* = 0.0001), for CA-125 + FASN was 76.9%, for CA-125 + GLS was 85.4% and for CA-125 + FASN + GLS was 87.7%. The highest AUC was obtained for the combined CA-125 + FASN + GLS (Fig. 1).

## Discussion

Several methods can be used to predict the results of cytoreductive operations, from the history-taking, physical examination, supporting examinations such as ultrasound, CT, MRI, PET, and laboratory [16–18]. Some studies found that CA-125 is not a sufficiently strong predictor for cytoreduction surgery [10, 19, 20]. In this study, the researchers aimed to assess other biomarkers, namely FASN and GLS, separately and in different combinations.

In his study, Memarzadeh et al. determined that CA-125 was still a very weak predictor for cytoreduction surgery because of its low sensitivity (58%) and a spesificity (54%) [21]. The cut-off point, 500 U/mL, used by Obeidat Basil, et al. was obtained from Chi’s study with stage III ovarian cancer patients as the samples [22], yielding a sensitivity of 72% and specificity of 73% for predicting suboptimal cytoreduction surgery by CA-125 [19]. However, in this study, the cut-off point value for CA-125 was 248.55 U/mL, with a sensitivity of 73.2%, spesificity of 73.6%, and accuracy of 73.3% (*p* = 0.0001).

CA-125, commonly referred to as MUC16, is a glycoprotein that protects the cell layer from external threats and supports the epithelial-mesenchymal transition, the essential process of metastasis in ovarian cancer [23, 24]. MUC16 has been identified as the most highlt expressed antigen in ovarian cancer [10].

Metabolic changes occur to supply proliferative cancer cells. These cells require the formation of adenosine triphosphate (ATP) in the fulfillment of their energy needs, which itself requires changes in the metabolic pathways of carbohydrates, proteins, lipids, and nucleic acids [25].

Lipids are molecules consisting of fatty acids and can be obtained exogenously and endogenously (de novo lipogenesis) [26]. These fatty acids are used to produce ATP in the proliferation of cancer cells, and the fatty acids produced by de novo lipogenesis protect cancer cells from free radicals and chemotherapy by forming the structure of cancer cell membranes [27]. These fatty acids are formed from the conversion of 12 of malonyl-CoA into palmitate with the help of FASN [28].

FASN has been shown to induce epithelial-mesenchymal transitions, thereby increasing the chances of ovarian cancer metastasis to the peritoneal cavity [29]. Therefore, tumours that have an aggressive phenotype will show increased FASN expression [30]. These results provided the basis for the researchers to pursue FASN as an alternative predictor to CA-125 for cytoreduction. However, we found that FASN was a weaker predictor than CA-125, having lower sensitivity. The combined FASN + CA-125 also did not seem to give better results than CA-125 itself, with a sensitivity of 71.4% and a specificity of 71.7% (*p* = 0.0001).

The primary source of nutrition that provides bioenergy to cancer cells for proliferation is glutamine [31]. The characteristic metabolic change in cancer cells is an increase in glutaminolysis. Glutamine is consumed by tumour cells ten times more than other amino acids because of its function in the proliferation and viability of most cancer cells [32]. Compared to that in healthy cells, the conversion of glutamine to lactate in cancer cells is incrased.[31] Because of its essential function in cancer cell metabolism, if glutamine deficiency occurs, cell growth inhibition can even induce apoptosis [31]. Yang et al. found that the level of cancer cell dependence on glutamine was highly correlated with the rate of cancer invasion [31]. Glutamine is metabolized through the Krebs cycle and influenced by 2 enzymes, namely GLS and glutamate dehydrogenase (GDHS) [13].

As a predictor of cytoreduction, GLS yielded a sensitivity of 73.2% and a specificity of 75.5% with a cut-off point of 22.895 (*p* = 0.0001). The combined GLS + CA-125 yielded better results than the individual biomarkers, with 82.1% sensitivity and 73.6% specificity. To the best of our knowledge, this is the first study to use FASN and GLS as predictors for cytoreduction surgery in EOC. Our study determined that GLS possesses a sensitivity and specificity almost equivalent to those of CA-125.

CA-125 had an accuracy of 73.3%, while GLS had an accuracy of 74.3%. Different combinations of biomarkers had increased sensitivity, specificity, and accuracy values in predicting cytoreduction. CA-125 + FASN + GLS had a higher accuracy (80.7%) than CA-125 + GLS (77.9%) or CA-125 + FASN (71.6%). Therefore, the clinicians could use this combination as an alternative to predict cytoreductive surgery in EOC.

## Conclusion

In summary, the individual roles of CA125, FASN and GLS levels in predicting suboptimal cytoreductive surgery for patients with EOC is questionable. However, the combination of CA-125 and GLS or CA-125, FASN and GLS can increase the sensitivity, specificity, and accuracy in predicting suboptimal cytoreductive surgery. The combined score is expected to help doctors to provide better therapy than before.

## Limitations

One of the limitations of this study was the use of stages II, III, and IV in epithelial ovarian cancer, as the staging of the cancer affected the tpe of cytoreductive surgery in this study (Table 1.). CA-125 is thought to be unable to predict cytoreduction because of its low value in mucinous-type ovarian cancer, but it is not known how other serum biomarker values such as FASN and GLS perform for this kind of cancer. The researchers were unable to assess the serum biomarkers in each category of epithelial ovarian cancer due to the lack of samples in each category.

## Data Availability

The datasets used and/or analysed during the current study are available from the corresponding author on reasonable request.

## Abbreviations

CA-125: Cancer antigen 125
EOC: Ephitelial ovarian cancer
FASN: Fatty acid synthase
GLS: Glutaminase
ROC: Receiver operating characteristic
AUC: Area under the curve
